# Umbilical cord-derived mesenchymal stem cells implantation on Hemivertebra defect with three-year follow-up: Biological approach in congenital scoliosis treatment - A case report

**DOI:** 10.1016/j.ijscr.2022.107602

**Published:** 2022-09-06

**Authors:** Ahmad Jabir Rahyussalim, Mochammad Kamal Nasser, Faiz Muhammad Al As'ady, Tri Kurniawati

**Affiliations:** a Department of Orthopaedic & Traumatology, Cipto Mangunkusumo National General Hospital and Faculty of Medicine Universitas Indonesia, Jakarta, Indonesia; b Stem Cell Medical Technology Integrated Service Unit, Cipto Mangunkusumo General Hospital, Jakarta, Indonesia; c Stem Cells and Tissue Engineering Research Cluster, Indonesian Medical Education and Research Institute (IMERI), Faculty of Medicine Universitas Indonesia, Jakarta, Indonesia; d Post Graduate Medical Doctor, Faculty of Medicine, Universitas Indonesia, Jakarta, Indonesia

**Keywords:** Umbilical cord-derived mesenchymal stem cells, Hemivertebra defect, Congenital scoliosis, Case report

## Abstract

**Introduction and importance:**

Congenital scoliosis is abnormal vertebral column growth and development during embryogenesis. The most common type of congenital scoliosis is failure of growth which is called as hemivertebra. However, the recent surgical treatment of hemivertebra has several complications especially in young patient. The mesenchymal stem cells (MSCs) have been used to treat several bone problems including bone defect and may be have potential to treat the defect in hemiverterbra. We reported a hemivertebra treated by umbilical cord-derived MSCs (UC-MSCs).

**Case presentation:**

A two-year-old boy presented with scoliosis deformity. The mother noticed the patient's deformity when he was 10th month of age as he learned to stand and progressed since then. There were no growth and development problems. On physical examination, the patient appeared to have scoliosis at lumbar level with bending to the right and asymmetry of waist fold with left shoulder depression. Based on X-ray and CT-Scan investigations, the patient was diagnosed with single fully segmented hemivertebra at 3rd lumbar level. 20 × 10^6^ UC-MSCs were implanted into the bone defect of hemivertebra.

**Clinical discussion:**

At three-year follow-up, the X-ray and MRI investigations showed a decrease of Cobb angle and increase of hemivertebra ratio. These findings may be due to improvement of the bone defect, which is consistent with several studies that MSCs have abilities to promote bone formation by maintaining the osteoblast cells and improving vascularization.

**Conclusion:**

We found that MSCs therapy for hemivertebra represent a potential therapy to correct scoliosis curvature and prevent further curvature. Further clinical studies are required to investigate the efficacy of this therapy in hemivertebra.

## Highlights

• Hemivertebrae is due to the failure of development of vertebrae.
• Mesenchymal stem cells induce new bone formation.
• Symmetrical growth of the spine is required for prevent spinal deformity.
• Regenerative therapy using mesenchymal stem cells as treatment strategy for hemivertebra
• Implantation of mesenchymal stem cells in hemivertebra is safe procedure.

## 1 Introduction

Congenital scoliosis is described as vertebral lateral deviation caused by abnormal vertebral column growth and development during embryogenesis. The worldwide prevalence of congenital scoliosis is estimated to be 0.5–1 per 1000 live births and the mortality rate is increasing in untreated cases due to cardiopulmonary problems [1], [2]. The involvement of genetic and environment factors in the disease's etiology has not been known. According to studies, developmental disorder occurs during spinal formation, called semitogenesis, in the third and fourth weeks of gestation [3]. These studies showed that deformity in congenital scoliosis can be distinguished into several groups, including failures of formation (hemivertebra), failures of segmentation (bar), and combination of both (mixed deformity). However, hemivertebra is the most common pathogenesis that produce asymmetrical spine growth in congenital scoliosis [4].

The treatment of congenital scoliosis is a challenge for orthopaedic surgeon. There have been no clear guidelines in respect to surgical treatment to correct the deformity especially in young patients. A study conducted by Weiss HR. (2016) concluded that there were no evidence to support the surgery in patients with congenital scoliosis is superior than no treatment or conservative treatment [1]. Moreover, the risk of neurological injury from surgical treatment in congenital scoliosis is higher than that of patients with idiopathic scoliosis [4].

In recent years, mesenchymal stem cells (MSCs) therapy has been established as one of the approaches used in the bone repair and regeneration process [5]. MSCs have the characteristics of self-renewal and pluripotency, as regenerative medicine, and exist in several post-natal tissues, such as adult bone marrow, adipose, and umbilical cord. Based on in-vitro and in-vivo studies, all of MSCs from different sources have the ability to differentiate into osteogenic and chondrogenic lineages thus promote osteoinduction and osteogenesis process [6]. The use of stem cells in bone repair and regeneration has raised many promises in orthopaedic problems, including congenital bone defect. The goal of this case is to know the effect of MSCs on the defect of hemivertebra. This case report has been written following the SCARE 2020 criteria [7].

### 1.1 Case presentation

**Fig. 1:**
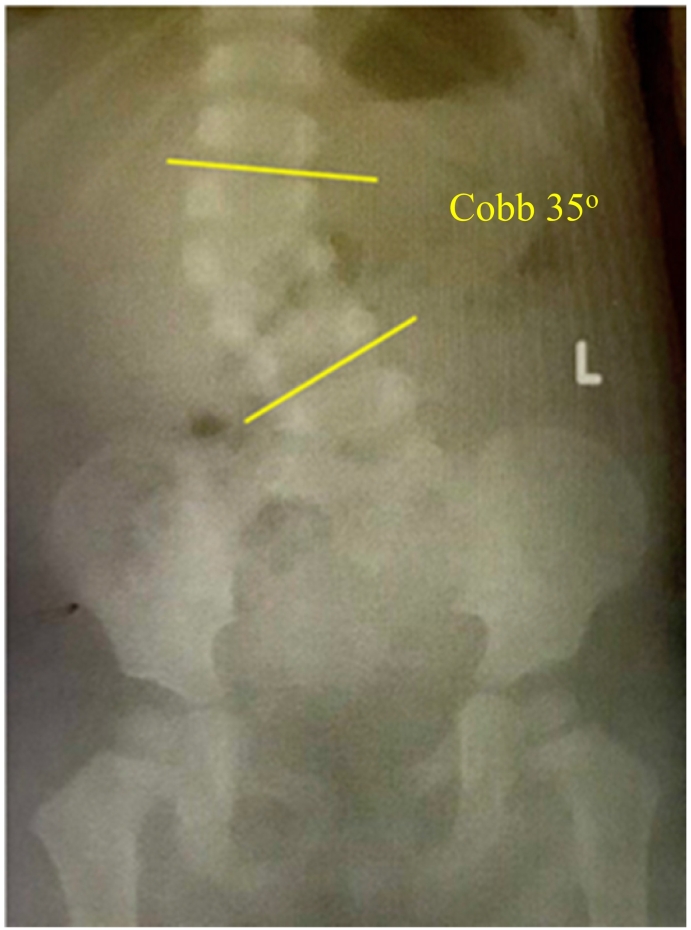
The X-ray of the patient before MSCs implantation showed hemivertebra on lumbar portion. The Cobb angle was 35 degrees.

**Fig. 2:**
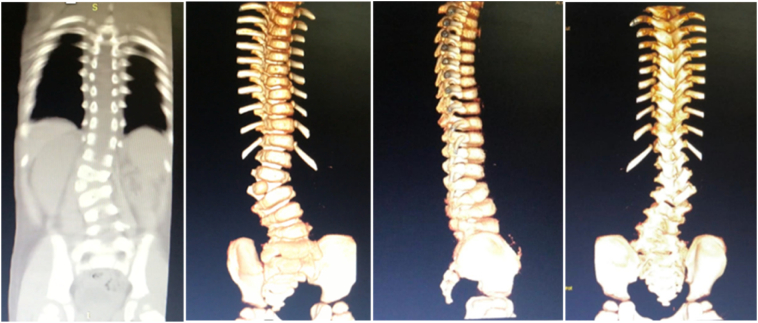
CT-scan and 3D CT-scan of the patient before MSCs implantation. A single fully segmented hemivertebra was visible at L-3 level.

A two-year-old boy was admitted to the bone and joint cluster with congenital lumbar scoliosis. The mother noticed the deformity at 10 months of age, as the baby learned to stand, and progressed since then. There was no growth and development problems. When the medical records of the patient were examined, it was reported that birth weight was 2800 g with aterm birth delivery. The mother said that there was no toxin exposure during pregnancy and she did not use any drug or cigarette. The boy and the parent had no other complaints and there was no history of scoliosis or genetic disorder in the family. On physical examination, the patient appeared to have scoliosis at lumbar level with bending to the right, asymmetry of waist fold with left shoulder depression. There was no neurological deficit in his upper and lower limb. The X-ray (Fig. 1) showed lumbar scoliosis with Cobb angle of 35 degrees of lumbar. The computed tomography (CT) scan (Fig. 2) revealed a single fully segmented hemivertebra at L-3 level.

The patient was not planned to undergo a surgery due to patient's age and the risk of surgical procedure. Instead, the parent had gone through an informed consent for MSCs implantation procedure. The MSCs were expected to grow and regenerate bone on the hemivertebra defect. The implantation was performed by orthopaedic team using C-arm guidance under general anesthesia in the operating room. With the patient pronation, 2 mm needle was inserted postero-laterally and 20 × 10^6^ umbilical cord-derived MSCs were implanted into the defect of hemivertebrae. Monitoring was carried out for possible side effects of treatment for 24 h post implantation. Allergic reaction, fever, pain and other side effects of the MSCs implantation were not found. After MSC implantation, patient can carry out normal activities as well as the activities of people of the same age.

### 1.2 Follow-up

**Fig. 3:**
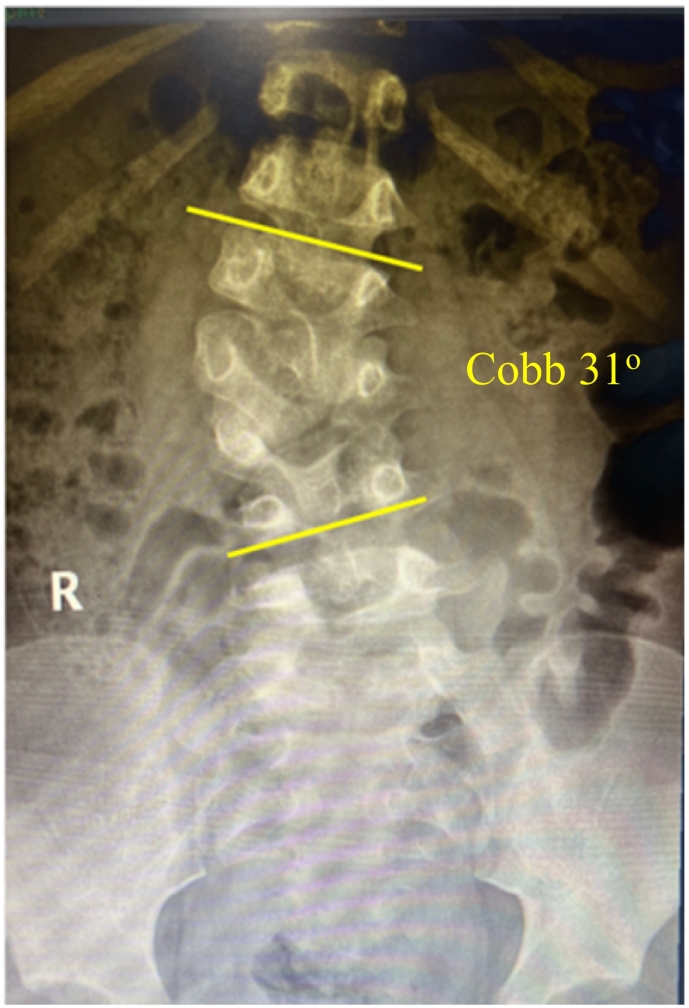
The X-ray 3 years post implantation showed lumbar scoliosis with Cobb angle of 31 degrees of lumbar.

**Fig. 4:**
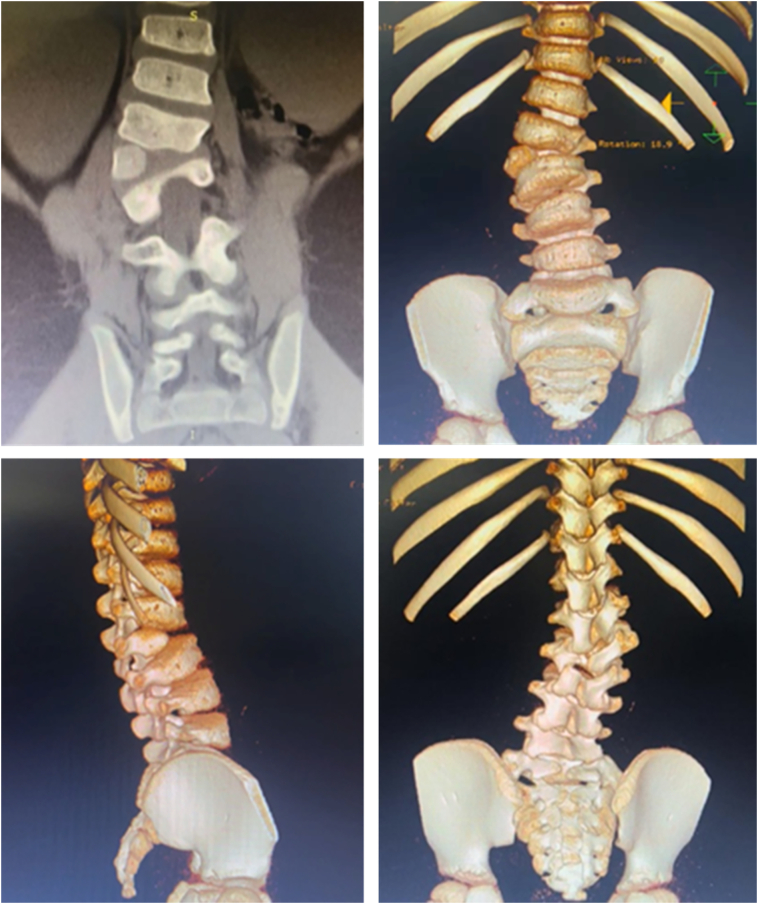
The CT-scan and 3D CT-scan 3 years post MSCs implantation. The CT showed a single fully segmented hemivertebra at lumbar level. There was no ectopic growth after implantation.

Because of COVID-19 pandemic, the patient did not come to the hospital and was loss to follow up after implantation. Three years later when the pandemic subsided, the boy and his parent were coming back to the hospital to check the current condition. There was no complaint that affected the patient's daily activities. He had no growth and development problem compared to other boys at his age (Five-year-old). On physical examination, the deformity still could be seen but had been improving compared to before implantation. We evaluated the X-ray and CT-scan. The X-ray (Fig. 3) showed improvement of lumbar scoliosis with Cobb angle was 31 degrees of lumbar (the Cobb angle before implantation was 35 degrees at the same level). The anatomy of the lumbar vertebra after MSCs implantation was revealed on CT-scan (Fig. 4).

**Fig. 5:**
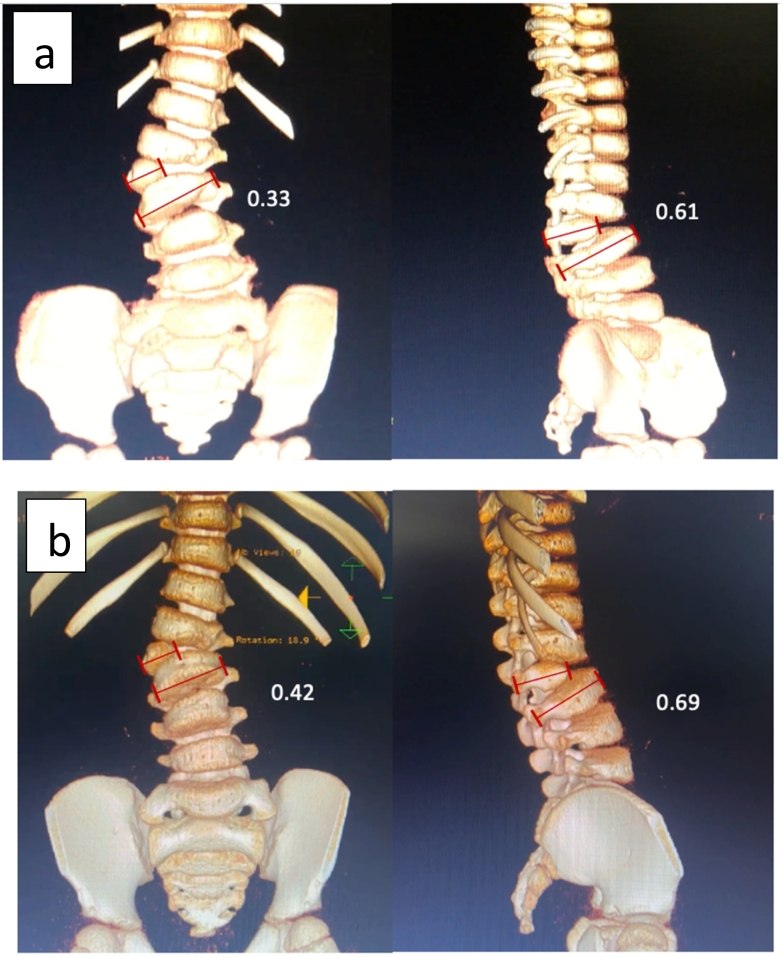
Comparison of 3D CT between a) before MSCs implantation and b) 3 years after MSCs implantation. The hemivertebra ratio was conducted from the maximum horizontal hemivertebra length and the maximum horizontal normal vertebra length (showed in red line). On the anterior view, the ratio increased from 0.33 to 0.42 whereas on the lateral view, the ratio increased from 0.61 to 0.69. (For interpretation of the references to colour in this figure legend, the reader is referred to the web version of this article.)

Fig. 5 showed the 3D CT-scan comparison between pre implantation and 3 years post implantation. The hemivertebra size was measured by conducting a ratio between horizontal hemivertebra length and horizontal normal vertebra body length on both anterior and lateral view. There was increasing ratio from 0.33 to 0.42 on anterior view and 0.61 to 0.69 on lateral view. The increasing of these ratio indicated the improvement of hemivertebra size horizontally.

## 2 Discussion

This is a rare congenital malformation of spine because the prevalence is estimated at 0.5–1 per 1000 births [1]. In our case, the affected patient was a boy and it is in line with studies conducted by Forrester MB et al. (2006) [8] that suggesting greater occurrence in males while another study conducted by Goldstein I et al. (2005) [9] reported equal incidence in both genders.

The pathogenesis of hemivertebra could be understood since the embryonic stage. Spinal development occurs during the sixth week of gestation when two lateral chondrification centers arising in the developing vertebral bodies. By 7–8 weeks of gestation, these chondrification centers fuse to form the primary ossification center of the vertebral body, which is temporarily divided into anterior and posterior aspects by notochord remnant. Lack of development of one of the paired chondral centers leads to lateral hemivertebra, whereas, less commonly, failure of anterior ossification center leads to posterior hemivertebra [9], [10], [11]. The defective vertebra causes contralateral spine deviation at the level of the abnormal vertebra by acting as a triangular wedge-shaped ossified structure within the vertebral column [12]. Four types of hemivertebra can be distinguished by the presence or absence of a normal disk space above and below the affected segment, i.e. fully segmented (most common type), semi-segmented, non-segmented, and incarcerated [1]. In this case, the malformation as shown on the CT-scan showed a fully segmented hemivertebra, as in agreement with the study from Bao B et al., fully segmented hemivertebra is the most common type among the others. However, fully segmented types of hemivertebra which has Y-shaped of disc in coronal view has potential for worsening because of asymmetric growth compared with other types [13].

The etiology of hemivertebra is unknown. An observational study conducted by Tanaka T hypothesized that hemivertebra may result from abnormal distribution of the intersegmental arteries of the vertebral column [14]. Hemivertebra can be isolated or may occur in multiple areas at any vertebral level and commonly associated with other congenital anomalies. Several skeletal anomalies of the spine, ribs, and limbs are usually associated with hemivertebra. Most common extramusculoskeletal anomalies seen in hemivertebra are cardiac and genitourinary tract anomalies. Nervous system and gastrointestinal tract anomalies were also reported in hemivertebra cases [15], [16]. In severe condition, hemivertebra may be a part of some genetic syndromes, such as Jarcho-Levin syndrome, Klippel-Fiel syndrome, and Vertebral anomalies, Anal atresia, Cardiac defects, Trecheooesophageal fistula and/or Esophageal atresia, Renal & Radial anomalies and Limb defects (VATER) syndrome [17]. Moreover, a study conducted by Song et al. (2016) concluded that hemivertebra is associated with 7q deletion with clinical manifestation of microcephaly, holoprosencephaly, facial anomalies, cardiac anomalies, currarino syndrome, growth and mental retardation, and short stature [18]. In our case, the patient had no growth, development, and mental problem and no urinary or bowel problem. He also had normal appearance compared with other children.

We treated our case with MSCs in order to grow and repair the bone defect of hemivertebra. After MSCs implantation, the patient was loss to follow up due to pandemic and came back 3 years later. We did the re-examination and considered it as long-term follow-up. At the follow-up, no complications occurred and there was no any sign of neoplasm formation, which is in line with previous studies that used MSCs to treat orthopaedic problems [6], [19]. Based on the X-ray, there was a decrease in Cobb angle of lumbar from 35 degrees before implantation to 31 degrees after implantation. This improvement could be explained from the CT-scan result which clearly show the anatomy of the vertebra. The hemivertebra size (based on the ratio between hemivertebra and normal vertebra) had increased compared to before implantation. The improvements were also occurred at the adjacent vertebra bodies. The wedge vertebra bodies above and below the hemivertebra had thickened and increased in volume. Furthermore, there was no ectopic growth of the vertebral bone and no spinal cord compression. These improvements change the scoliosis curvature as a whole. This is in line with some studies that explained the MSCs have abilities to migrate into the defect area then stimulate osteogenesis [20], [21]. The osteogenesis need a good environment to support its process including a normal vascularization. MSCs have the ability to induce angiogenesis and neovascularization through VEGF secretion which is expected to repair the abnormal distribution of intervertebral arteries in hemivertebra [14], [22].

Regenerative medicine using MSCs has become a potential treatment strategy for bone problems, which has great deleterious effect on patient's daily activities and society [23]. Bone defect, as one of bone problems, is commonly caused by trauma, tumor resection, surgery, infections, and also congenital malformation [24]. In some conditions such as pathological fracture or large and massive bone defects, the abilities of bone to repair itself are fail, hence, bone regeneration with clinical intervention is needed. Moreover, bone repairing capacity can be affected by several conditions including bone infection, systemic diseases, and insufficient blood supply, as the one of the hypotheses of hemivertebra etiology [14], [24].

MSCs have abilities to increase osteoinduction and osteogenesis. These cells contribute significantly to bone regeneration and repair through a variety of processes including providing cell migration, homing, angiogenesis, anti-inflammatory effect, and differentiation [25]. In MSCs-based therapy, the fundamental steps for bone formation and defect repair are the ability of migration and homing into injured or defect areas. The recruitment of MSCs is triggered by the response of MSCs to inflammatory factors released from the injured area, these processes are mediated by interaction between chemokines, chemokine receptors, adhesion molecules, and proteases [20], [26]. Implanted MSCs support bone regeneration through angiogenesis stimulation by producing hypoxia inducible factor 1 alpha (HIF-1⍺) in response to hypoxic perivascular niches. The HIF-1⍺ then stimulates the angiogenic factors such as vascular endhotelial growth factor (VEGF), transforming growth factor beta (TGF-β), stromal cell-derived factor-1 (SDF-1), and stem cell factor (SCF) [27]. The VEGF plays an important role in angiogenesis and neovascularization in bone development [22]. In an autocrine manner, MSCs can express bone morphogenetic protein-2 (BMP-2) in defect bone, thus promoting the differentiation of these cells into osteoblasts [28], [29]. BMP-2 is an important protein in bone healing due to its involvement in bone tissue formation, increasing osteoblast function, and maintaining the dynamic balance of the new bone tissue [21]. BMP-2 involves in osteogenesis by interacting with BMP-2 receptors thus stimulates two signal pathways including Smad-dependent pathway and the mitogen-activated protein kinase (MAPK) pathway [30], [31]. MSCs have also become an important role in proliferation of chondrocytes. There are two main mechanisms for MSCs involve in cartilage improvement: a) prevent degradation of cartilage by secreting bioactive factors, such as tissue inhibitor of metalloproteinase 2 (TIMP-2) and TIMP-1 inhibitors, thus suppress cartilage destruction, and b) By releasing growth factors, cytokines, and signaling molecules, such as TGF-β1, TGF-β2, TGF-β3, Wnt/β-catenin signaling pathway, Thrombospondin (TSP2), thus promotes MSCs differentiation to chondrocytes [32], [33].

MSCs implantation is safe and has been used clinically to treat bone problems, such as osteoarthritis, intervertebral disc degeneration, osteoporosis, and congenital deformity [6].

This case serves as MSCs treatment strategy for congenital scoliosis.

## 3 Conclusion

We found that MSCs therapy for hemivertebra represent a potential therapy to correct scoliosis curvature and prevent further curvature. Further clinical studies are required to investigate the efficacy of this therapy in hemivertebra.

## Sources of funding

Financial support was provided by 10.13039/501100006378Universitas Indonesia for funding this research through PUTI Grant Universitas Indonesia (BA-1054/UN2.RST/PPM.00.03.01/2020) for the research, authorship, and/or publication of this article.

## Ethical approval

Not applicable.

## Consent

Written informed consent was obtained from the patient for publication of this case report and

accompanying images. A copy of the written consent is available for the review by the Editor-in-Chief of this journal on request.

## CRediT authorship contribution statement

Ahmad Jabir Rahyussalim: Supervising, making the concept and design of the treatment

strategy, analyzing the data

Mochammad Kamal Nasser: Collecting the data, analyzing the data, writing the manuscript

Faiz Muhammad Al As’ady: Collecting the data, analyzing the data

Tri Kurniawati: Editing the manuscript

## Research registration

Not applicable.

## Guarantor

Ahmad Jabir Rahyussalim.

## Provenance and peer review

Not commissioned, externally peer-reviewed.

## Declaration of competing interest

The authors declare that there is no conflict of interest regarding the publication of this paper.
